# Human Activity Recognition Algorithm with Physiological and Inertial Signals Fusion: Photoplethysmography, Electrodermal Activity, and Accelerometry

**DOI:** 10.3390/s24103005

**Published:** 2024-05-09

**Authors:** Justin Gilmore, Mona Nasseri

**Affiliations:** 1 Department of Electrical and Computer Engineering, University of Central Florida, Orlando, FL 32816, USA; 2 School of Engineering, University of North Florida, Jacksonville, FL 32224, USA

**Keywords:** human activity recognition, multi-modal classification, accelerometer, blood volume pulse (BVP), electrodermal activity (EDA), machine learning, convolutional neural network (CNN)

## Abstract

Inertial signals are the most widely used signals in human activity recognition (HAR) applications, and extensive research has been performed on developing HAR classifiers using accelerometer and gyroscope data. This study aimed to investigate the potential enhancement of HAR models through the fusion of biological signals with inertial signals. The classification of eight common low-, medium-, and high-intensity activities was assessed using machine learning (ML) algorithms, trained on accelerometer (ACC), blood volume pulse (BVP), and electrodermal activity (EDA) data obtained from a wrist-worn sensor. Two types of ML algorithms were employed: a random forest (RF) trained on features; and a pre-trained deep learning (DL) network (ResNet-18) trained on spectrogram images. Evaluation was conducted on both individual activities and more generalized activity groups, based on similar intensity. Results indicated that RF classifiers outperformed corresponding DL classifiers at both individual and grouped levels. However, the fusion of EDA and BVP signals with ACC data improved DL classifier performance compared to a baseline DL model with ACC-only data. The best performance was achieved by a classifier trained on a combination of ACC, EDA, and BVP images, yielding F1-scores of 69 and 87 for individual and grouped activity classifications, respectively. For DL models trained with additional biological signals, almost all individual activity classifications showed improvement (*p*-value < 0.05). In grouped activity classifications, DL model performance was enhanced for low- and medium-intensity activities. Exploring the classification of two specific activities, ascending/descending stairs and cycling, revealed significantly improved results using a DL model trained on combined ACC, BVP, and EDA spectrogram images (*p*-value < 0.05).

## 1. Introduction

With recent technological advancements in developing multi-sensor wearable devices, research related to physiological signals has grown rapidly. Classifying human physical activities has recently received a lot of attention due to its connection to physical and mental health. Physical activity (PA) is not only essential for preventing obesity; it might also confer neuroprotection in Alzheimer’s (AD), Parkinson’s (PD), and Huntington’s (HD) diseases via the upregulation of synaptic signaling pathways [1]. It also helps in rehabilitation processes such as cardiorespiratory fitness (CRF) [2]. Wearable physical activity (PA) monitoring systems have also been developed for monitoring the activity levels of a specific population, such as athletes or elderly individuals. E-textile sensors combining accelerometer and goniometer data assist in estimating joint angles; smart insoles are utilized for gait analysis; and smart garments are used in measuring respiratory rate during PA [3,4,5]. In general, knowledge of the frequency and intensity of an individual’s activity plays a significant role in improving his or her lifestyle, which is achievable using a reliable classifier.

Several factors must be considered in improving HAR classification performance, including the placement of wearable [6,7,8,9]. Using data acquired from the wrist is challenging due to the often non-activity-related natural hand/wrist movements. However, the wrist is the recommended collection site for increased wear time [7,8].

Among physiological signals, accelerometer (ACC) data are the main signal in proposed algorithms. In a recent survey of 163 selected HAR studies, it was shown that 149 deployed at least one accelerometer or an accelerometer in conjunction with another sensing modality (gyroscope, magnetometer, body temperature sensor, electrocardiograph, electromyography, etc.) [10]. The prevalent use of accelerometers in HAR may be explained by the low cost and small size of the device as standalone sensors, and additionally, their low energy consumption and high feature performance, as demonstrated in modern smartphones, further contribute to their widespread adoption [11,12].

Recently, the concept of multi-modal HAR has been explored, and it was shown that multi-modal-based methods can improve classification results by leveraging the complementary characteristics between the single modes [13]. For example, accelerometers were used in conjunction with other sensors including gyroscopes [14], magnetometers [15], heart rate (HR) and respiration rate sensors [16], electrocardiographs [17], surface electromyography (sEMG) [18], and skin conductance measurement (EDA) [19].

Jia and Liu [20] proposed a classification method to harness data from a 7-lead ECG worn on the chest and an accelerometer magnitude vector recorded from waist. Time- and frequency-domain features were calculated for classifying lying, sitting, standing, walking, walking upstairs, walking downstairs, and running activities. Average classification accuracy from accelerometer data alone was 93.83%, and it was increased to 99% when fused with ECG data.

Photoplethysmography (PPG) is a less-obtrusive alternative to ECG for obtaining a continuous cardiac signal. It was also shown that ECG signals can be reconstructed from PPG signals [21]. Furthermore, advancements in optical sensors have made PPG an alternative for measuring pulse rate variability features, as surrogates for heart rate variability (HRV) indexes [22]. These techniques offer greater convenience and less intrusion compared to traditional methods.

The derived signal from the PPG sensor is called the blood volume pulse (BVP), and it is the measurement of changes in blood volume in arteries and capillaries. Interestingly, changes in the BVP signal can reflect changes in blood pressure [23], which may correspond to levels of exertion characterizing some types of activities [24]. These attributes make it a promising modality for a wide array of applications, including but not limited to human activity monitoring [17,25,26,27].

However, classifier performance improvement related to non-ACC signal data may be activity-dependent. The use of accelerometer data alone can be insufficient for recognition of activities that require little movement but considerable energy consumption [28]. An example is an activity such as cycling, when a wrist-worn accelerometer is measuring mostly the slight movements of the wrist. Interestingly, a study conducted by Weippert et al. demonstrated that heart rate variability measures could be used to distinguish static exercise (supine leg press) from dynamic exercise (cycling) when the heart rate for each was similar [29]. Another study shows that combining selected HRV features from a single-lead ECG resulted in improved performance in separating sitting from standing and walking from ascending-walking classes over that obtained from accelerometer alone [28]. ECG-derived signals such as PPG data, although not proven to be informative enough on its own as an effectively discriminating input, have provided the capability to improve the separation of activities (when combined with a triaxial accelerometer) involving similar movement yet requiring different levels of exertion, such as cycling and cycling with added resistance [30].

A recent study employed an ensemble method of pre-trained deep learning models (Resnet50V2, MobileNetV2, and Xception), fused at the feature level, to classify four activities (running, walking, and high/low resistance cycling), for the purpose of comparing the efficacy of PPG input data to ECG [25]. The resulting accuracies of the ensemble model tested on the ECG and PPG data were around 94% and 89%, respectively, demonstrating a potential significant improvement in classification accuracy when PPG is used in deep learning models (CNNs) over machine learning models.

Recording a high-quality PPG/BVP signal is complex, since it is influenced by changes in ambient light, electrical noise from the device, and sensor movement [26]. Methods to alleviate the motion artifact (MA) contained in PPG signals due to physical activity have been proposed at the cost of increasing complexity to the signal processing phase of activity classification [31,32]. However, the PPG signal’s susceptibility to corruption by noise introduced by MA has been exploited to predict activities [27].

Another additional signal that has the potential to improve HAR performance is electrodermal activity (EDA). EDA represents changes in the electrical conductivity of the skin. The most salient characteristic of an EDA signal is the skin conductance responses (SCRs) resulting from an underlying sympathetic reaction to a stimulus [33]. The SCRs are the rapid-onset and smooth, exponential-like, transient events noticeable in the EDA signal. EDA data have been used in the prediction of seizures [34], human emotion recognition [35], menstrual cycle analysis [36], and in affective computing [37]. Somewhat recently, EDA has been used in the classification of activity intensity as perceived by the subject (relative PA intensity) [19,38].

Poli et al. [19] trained SVM and bagged tree models on features taken from triaxial accelerometry, HR, inter-beat interval (IBI), skin temperature, and EDA. Using the bagged tree classification model trained on EDA data alone resulted in an average F1-score of 73.8%—which increased to 93.9% when all modalities were included—for classification of sedentary, moderate, and vigorous activities.

Despite the popularity of classifying physical activities using time-series data, some studies focused on using image representation of activities. Such a classification system typically employs a CNN for class prediction. Short-time Fourier transform (STFT), reduced interference distribution with Hanning kernel (RIDHK), and smoothed pseudo Wigner–Ville distribution (SPWVD) have been used in the classification of radar echo signals produced by various physical activities with averaged accuracy (over six physical activities) of 96.61%, 94.72%, and 91.06%, respectively [39]. Accuracy was increased to 98.11% when all three images were vertically concatenated and used as input to a VGG16 network.

In another study, accelerometer and gyroscope data from the Sussex-Huawei Locomotion (SHL) dataset [40] were used to generate FFT spectrograms as inputs to a NN classifier [41]. The network was trained separately on spectrograms corresponding to each axis of both sensors resulting in F1-scores of 90.5%, 91.1%, 90.1%, and 92.8% for accelerometer spectrograms (x, y, z, and magnitude, respectively) and 84.7%, 87.8%, and 83.7% for gyroscope spectrograms (x, y, and z axes, respectively). It should be noted that the SHL dataset contains certain activity classes (riding in a car, bus, train, and on the subway) which are not human physical activities per se.

This study aims to explore the significance of non-inertial signals in enhancing the recognition of human activities within both feature-based and deep learning classifiers. The subsequent sections delve into the processes of collecting physiological data, preprocessing signals, extracting features, generating spectrogram images, and employing classification techniques. These methods are then evaluated against inertial-based classifiers to assess their performance. Furthermore, the impact of blood volume pulse (BVP) and electrodermal activity (EDA) signals on enhancing the recognition of cycling and stairs-climbing activities is investigated and reported.

## 2. Materials and Methods

**Figure 1:**
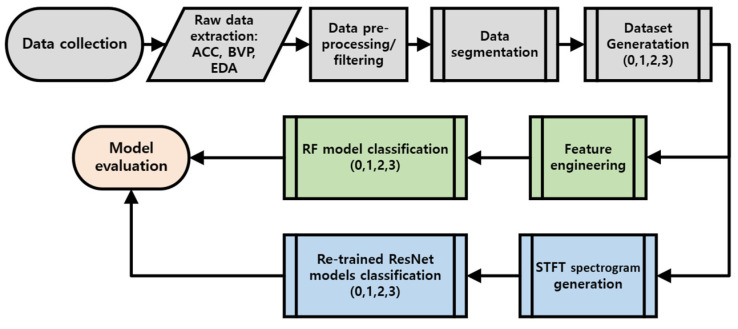
Flowchart for HAR algorithm development; ACC, BVP, and EDA data were collected from a wristband device. RF algorithms were trained on features extracted from signals, and DL algorithms were trained on spectrogram images.

The interest of this study is in determining whether a classification system can be improved by the fusion of multiple physiological inputs with triaxial ACC data. The performances of a random forest (RF) classifier and a pre-trained deep learning (DL) model (ResNet18) were analyzed using combinations of physiological signals. RF models were trained on features, and DL models were trained on short-time Fourier transform (STFT) images, engineered from the following modalities: 0-ACC, 1-ACC, and BVP; 2-ACC and EDA; and 3-ACC, BVP, and EDA signals. These datasets will be referred to as 0, 1, 2, and 3, respectively, and the corresponding classification models will be referred to as C0, C1, C2, and C3 for both RF and DL classifications. Workflow of the HAR algorithm development—demonstrating its general steps, including data collection, data processing, dataset generation—and the two approaches that were taken in this paper to develop RF and DL algorithms are shown in Figure 1. Each step is described in detail in the following sections.

### 2.1. Physiological Data Collection

In this study, the Empatica E4 (Empatica Inc., Boston MA) wristband was used to collect physiological data from subjects while performing a set of activities. E4 sensors include (1) a MEMS-type triaxial (x, y, and z) accelerometer that measures continuous gravitational force on the scale ±2 g with sampling frequency of 32 Hz; (2) a PPG sensor, from which BVP and HR data are derived (sampling frequencies of 64 Hz and 1 Hz, respectively); (3) an EDA sensor recording data at a sampling frequency of 4 Hz; and (4) a skin temperature sensor recording data at a sampling frequency of 4 Hz [42].

An Institutional Review Board protocol (#1796115) was approved for data collection at the University of North Florida and University of Central Florida. Twenty-three subjects (20.7 ± 1.6 years old), including ten females and thirteen males, were recruited to wear the wristband during the experiment. Before starting the recording session, subjects were provided with all information regarding this study, as well as the consent form to review and sign. The subjects were helped to set up the wristband and to ensure the proper placement. The device was worn on the non-dominant wrist. A fifteen-minute time duration was considered for the device to warm-up before starting the session. Subjects were asked to perform a series of activities, each of five-minute duration or—if less—for a duration that the subject was comfortable performing a given activity. Subjects were instructed on how to perform each activity; however, to simulate free-living conditions, there were no strict requirements defining each activity. A short break between activities was given for rest, hydration, and to allow the subject’s heart rate to return to as close to his/her baseline as possible. The baseline heart rate was obtained during the sitting activity. The activities were performed in the following order: sitting, standing, lying, walking, brisk walking, jogging, running (on the indoor track), cycling (stationary bicycle at a rate of around 80 revolutions per minute without resistance), and using stairs (ascending and descending), with the non-dominant hand free to swing. The total data recorded (756.25 min) consisted of 112.95, 110.27, 110.2, 105.81, 91.25, 122.63, 103.14, and 121.42 minutes in brisk walking, cycling, jogging, lying, running, stairs, standing, and walking, respectively.

All activities were performed in the University of North Florida Student Wellness Complex or the University of Central Florida Recreation and Wellness Center. All the activity start times were labeled by tapping the E4’s button (<1 s), and the type of activity was recorded by a proctor. The timestamps later were extracted from a downloaded .csv file that contains a record of the times of the events marked during a session.

### 2.2. Data Processing

Upon completion of the activity sessions, the subject’s data were inspected via the E4 Connect session viewer. Event markers denoting the start and stop time of each activity performed were verified against the times marked by hand in the session. The data corresponding to the first and last five seconds of an activity were discarded along with occasional segments of the recording during which it was noted that the subject either momentarily stopped performing the activity or the subject’s wrist movement (sensor-worn wrist) became excessive (for example, during standing or lying).

For each session, a timestamp series was generated, determining the start and end time of each activity. For data segmentation, initially, window sizes ranging from 5 to 20-second with a step size of 5-second were generated and tested. Ultimately, we selected a 10-second window size to ensure sufficient frequency resolution for electrodermal activity (EDA) features and to improve overall performance. Additionally, a 50% overlap was used to achieve both an informative window size and acceptable dataset size [43].

All pre-processing steps (filtering and segmentation) and feature computations were completed using Python v. 3.8.11. A second-order Butterworth bandpass filter (0.3–10 Hz) was used to remove the DC component and frequencies above 10 Hz in ACC data (x, y, and z components), noting that 98% of the spectral amplitudes corresponding to human activities lie below 10 Hz [44]. PPG and EDA signals were filtered using a fourth-order Chebyshev-II band pass filter (0.4–5 Hz [45]), and a fourth-order Butterworth high-pass filter (0.05–2 Hz), respectively. Datasets 0, 1, 2, and 3 were generated from the processed and segmented data for all subjects. Time- and frequency-domain features computed for RF classification are listed by modality in Table S8. STFT images were generated from 10-second data segments of BVP, EDA, and ACC magnitude, with a 50% overlap.

### 2.3. HAR Classifier Development

A HAR system which included two independent algorithms was assembled. First, a random forest algorithm—which is among the popular classification algorithms for HAR models [17,46]—was trained on features extracted from 10-second data segments. Then, as a DL classifier, a convolutional neural network obtained via transfer learning was trained on spectrogram images of 10-second pre-processed ACC, BVP, and EDA data segments.

#### 2.3.1. Random Forest Algorithm

The RF classifier from the sklearn.ensemble module with 500 estimators (trees) was used in this study. Grid search was performed over a range of 100 to 1000 with a step size of 100 to optimize this hyperparameter. The remaining default parameters were used, including min_samples_split of 2, and max_feautres of sqrt. Four RF algorithms (C0, C1, C2, and C3) were trained to classify eight individual activities (lying, standing, walking, brisk walking, jogging, running, stairs, and cycling) using features from datasets 0 through 3, respectively. Next, activities were grouped according to the body movement involved in the activity and activity–intensity overlap, which may have likely been induced by the relaxed experimental environment. Therefore, standing and lying, walking and brisk walking, and jogging and running engendered low-, medium-, and high-intensity groups. Cycling and stairs remained separate activity classes. RF algorithms C0 through C3 were also trained on grouped-activity datasets.

Features, both low-level and handcrafted, extracted from ACC, BVP, and EDA data, are reported in Table S8. The feature set is represented by statistical estimators, such as those used in previous HAR studies [17,19,47], and some experimentally and intuitively derived [48]; for example, a range of frequency bands associated with respective levels of cardiac output.

Training and testing were conducted in leave-one-subject-out mode. Training data were further divided into 90%/10% randomized train/validation datasets. Data were then normalized (z-score, sklearn.preprocessing.StandardScaler), and a single test was conducted for each test subject.

#### 2.3.2. Convolutional Neural Network (CNN)

Pre-trained DL image classification neural networks eliminate the laborious task of designing a DL network and provide a re-trainable model which has learned rich feature representations from more than a million images. MATLAB’s (R2021b) Deep Learning Toolbox Model for ResNet-18 [49] was used in this study to perform the DL experiments (individual activity classification and group-based classification), analogous to the machine learning experiments described in Section 2.3.1.

**Figure 2:**
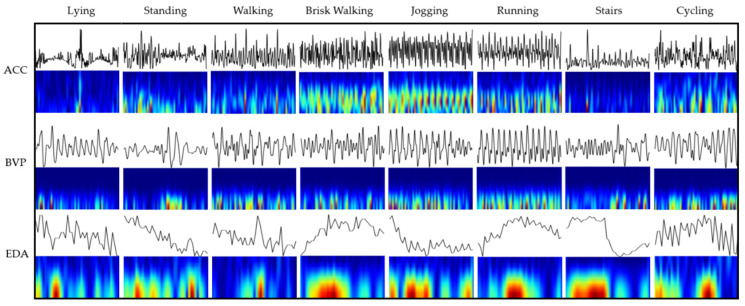
Paired time and time–frequency plots (STFT) of each activity corresponding to their ACC, BVP, and EDA segments (top to bottom) from a 20-year-old female subject. Activities represented are lying, standing, walking, brisk walking, jogging, running, stairs, and cycling (left to right). The colors in spectrogram represents the amplitude of a specific frequency at a given time, ranging from dark blues for low amplitudes and dark red, indicating stronger amplitudes.

STFT images were generated from datasets 0 through 3. The magnitude vector of ACC segments (i.e., ⅓ sqrt(ACCx^2^ + ACCy^2^ + ACCz^2^) was used to construct STFT images for dataset 0. Datasets 1 through 3 were created through vertical concatenation of the STFT images. The original ACC and EDA image size was 220 × 220 pixels; however, the BVP spectrogram image was cropped to a 220-width-by-110-height size due to the excessive near-zero-valued amplitudes present in the STFT higher frequencies. After vertical concatenation, the combined image was resized to 224 × 224, as was required by the ResNet-18 input shape. New classification and fully connected layers were created, and they replaced the two last ResNet-18 network layers to reduce the number of outputs from one thousand to eight classes. No other changes were made to the network. Time series signals are shown in Figure 2, along with their spectrograms, for eight activities.

The initial learning rate and batch size were set to 0.00125 and 75, after implementing a hyperparameter grid search over the ranges of 0.0005 to 0.002 with step size of 0.00025 and 25 to 125, with step size of 0.00025, respectively. Training data were divided into 90%/10% randomized train/validation datasets. All data were normalized, applying zero-center normalization, which normalizes each pixel value between [–1, 1]. Classifiers were trained for 50 epochs using the Adam optimizer.

## 3. Results

Data from twenty-three subjects performing eight activities (lying, standing, walking, brisk walking, jogging, running, ascending/descending stairs, and cycling) were included in the analysis of HAR classifier performances. First, individual activity classification was conducted. Then, for a less-granular classification task, six of the eight activities were grouped by their similar intensity (standing/lying (low), brisk walking/walking (medium), and jogging/running (high)). RF and CNN algorithms were trained to classify activities at both individual and group levels. Classifiers were trained on ACC (C0); ACC and BVP (C1); ACC and EDA (C2); and ACC, BVP, and EDA (C3) datasets. CNN was trained on STFT spectrogram images, while RF was trained on featurized data. To assess and compare classifiers performance, the area under the ROC curve and F1-score were calculated. Table 1 presents the performance of each classifier by input, both for individual and grouped activities. Due to variation in CNN model prediction from trial to trial, the average of three CNN trials is reported.

The results show that the RF classifier outperforms CNN; however, EDA and BVP signals improve the performance of the CNN classifier. AUC-ROC and F1-scores are reported in Table 2, Table 3, Table 4 and Table 5 for individual and grouped activity classification. Additional signals do not appear to improve RF classification results in individual or grouped activities (Table 2 and Table 4); however, for the CNN model, nearly all individual activity classifications were improved with additional signals (Table 3). In grouped activity classifications, CNN performance was improved for low- and medium-intensity activities, as well as for cycling and stairs (Table 5).

To determine if the improvement in performance was significant, a comparison of the three signal-augmented models to a baseline ACC-only CNN model was conducted using a mid-*p*-value McNemar test at the 5% significance level. The McNemar test showed statistically significant improvement in the classification results (*p* < 0.05) of C3 for all classes (as is shown in Table 3 and Table S2), except running, when compared to C0. Statistically significant improvement (*p* < 0.005) was also observed in grouped low- and medium-intensity stairs and cycling activities for C1, and in cycling for C2 when compared to C0 (Table 5 and Table S3). Adding both EDA and BVP inputs (C3) improved the performance of classification of all groups significantly (*p* < 0.005), except high-intensity activities.

Among common activities [10], stationary cycling and ascending/descending stairs appear to be two of the more difficult classes to discriminate from other modes of ambulation—or, in the case of cycling, from sedentary activities such as sitting—when using accelerometer data acquired from the wrist [7,17,50,51]. At a granular individual activity classification level, a Wilcoxon rank sum test was conducted to compare the classification accuracy of both cycling and stairs by comparing signal-augmented models to a baseline ACC-only CNN model. In C3, misclassification of stairs as standing and walking activities (*p* < 0.05) and cycling as lying and standing activities (*p* < 0.005) was significantly improved (Tables S4 and S5). Misclassification of cycling as standing was also decreased using C2 (*p* < 0.05). Misclassification of stairs and cycling with respect to the rest of the activities was negligible.

For grouped activity classification, a similar rank sum test was conducted. Using C3, misclassification of stairs as a low-intensity activity group (*p* < 0.05) and cycling as low- and medium-intensity activities (*p* < 0.005 and *p* < 0.05 respectively) was reduced. The combined ACC and BVP inputs improve classification error rates of both cycling and stairs, which were misclassified as low-intensity activities by C0 (Tables S6 and S7). Misclassification of stairs and cycling with respect to other activities not mentioned above was negligible.

## 4. Discussion and Conclusions

The application of wearable sensors in HAR system development continues to enjoy multi-disciplinary interest due to its importance in the monitoring of human well-being. Although most of the available algorithms rely on accelerometer sensor data, the purpose of this paper is to investigate the importance of BVP and EDA signals in HAR algorithms. Therefore, a multi-modal sensing wristband was employed for data collection. It should be noted that other signals, such as EMG and ECG, have the potential to improve PA classification results; however, the purpose of this study was limited to the data derived from signals obtained from a single Empatica E4 wristband.

Data from PPG, EDA, and triaxial ACC sensors were preprocessed and used to train machine learning and deep learning classifiers, with input modalities including only ACC data (C0); ACC and BVP (C1); ACC and EDA (C2); and ACC, EDA, and BVP (C3) to examine the importance of these non-ACC signals.

No significant improvement was observed in the RF classifier by adding additional signals. However, significant improvements were observed in overall F1-scores for CNN models trained on additional bio-signals and were confirmed by applying a McNemar test (*p* < 0.05). In individual activity classification, the average CNN F1-score was improved from 64.22% with C0 to 69.01% with C1, 67.7% with C2, and 69.89% with C3. The corresponding F1-scores for group classification also were improved from 80.65% with C0 to 86.34% with C1, 83.77% with C2, and 87.51% with C3. Regarding individual activity classification, a mid-*p*-value McNemar Test showed that performance of C3 was improved over C0 for all activities except running (*p* < 0.005); performance of C1 was improved over C0 for standing, lying, cycling, and stairs (*p* < 0.05); and performance of C2 was improved over C0 for cycling, jogging, lying, and stairs classes (*p* < 0.05). For the grouped, five-activity classification task, the McNemar Test showed that performance of both C1 and C3 was improved over C0 for all grouped activity classes except high-intensity (*p* < 0.005); and the performance of C2 was improved over C0 for cycling class (*p* < 0.05). This may suggest that there is a link between improved classification of cycling activity and EDA data facilitated by minimum wrist movement and, consequently, lower motion artifact. It is shown in Table 1 (also Table S1) that training the CNN model on BVP and/or EDA inputs consistently improves the average F1-scores, Matthews Correlation Coefficient (MCC), and Cohen’s kappa score.

Although there is no significant improvement in classification by a feature-based classifier such as the RF trained on bio-signal features, statistically significant improvement in classification by a multi-modal CNN model was observed. This improvement might be due to the ability of DL models to directly learn robust features from raw data for specific applications, whereas ML models such as RF require expertly extracted or engineered features for satisfactory performance [52]. Specifically for EDA and PPG, which are susceptible to noise, statistical features are less suitable. Other ML classification challenges may be (1) the issue of “overlapping” statistical characteristics of time- and frequency-domain signals, corresponding to certain activities (e.g., running and jogging) in free-living conditions (reduction of inter-class variability); (2) that for time spent in activity, even one as moderate as walking, cardiac output increases initially over a period of time and then tends to plateau [53]. This would imply that the beginning and end of a single activity may vary significantly in bio-signal amplitudes.

Motion artifacts also affect the performance of multimodal classifiers. In the BVP signal, the main artifacts composed of wrist acceleration in the x, y, and z directions and the changes in space between the skin and the PPG sensor, if considerably regular or periodic, are likely correlated with motion-intensity, which may characterize the subject’s activity. A spectral representation of a PPG signal has a dominant quasi-periodic signal component resulting from rhythmic wrist movement/foot–ground contact, and another component corresponding to heartbeat. If the two components have close periods, due to leakage, the spectral peak associated with the heartbeat may not be distinguishable from the peak associated with the wrist swing rhythm [26]. This condition may not be helpful to ML models but could be exploited by DL models.

The findings of our study, regarding the improved classification performance of a CNN despite being trained on MA-corrupted BVP inputs, may be supported by conclusions from research which used a combined convolutional and recurrent layer network to classify standing, walking, jogging, jumping, and sitting classes [32]. In this study, each subject’s PPG signal was decomposed into a cardiac component (0.5–4 Hz), a respiratory component (0.2–0.35 Hz), and a MA noise component (≥0.1 Hz) and converted to the frequency-domain using the FFT. When comparing models trained on data composed of the cardiac, respiratory, and MA components (individually and in combination), results suggested that the MA signal is a better predictor than the cardiac and respiration signal. Further, the predictability of activities from PPG does not only result from the elevation of heart rate and respiration rates but also from the MA generated when performing the activities [32]. Results from the C1 classifier in our study might suggest that the added BVP data could make classification by DL models robust against MA, given that the model is trained on noisy BVP data yet model accuracy significantly improves.

Among common activities in ML HAR datasets [10], stationary cycling and ascending/descending stairs appear to be more difficult to discriminate from other activities when using accelerometer data acquired from the wrist [7,17,50,51]. A somewhat heavily MA-affected activity, ascending/descending stairs is mostly misclassified as walking using ACC-based classifiers. However, the CNN model trained on BVP, EDA, and ACC data (C3) demonstrated significant improvement in separating stair use from walking (*p* < 0.05). On the other hand, cycling, which may be less affected by MA than ascending/descending stairs, is commonly misclassified as a sedentary activity such as sitting or standing. Results from our study show that the cycling class becomes more separable from lying and standing (*p* < 0.005) for CNN model C3. These results may point to a potential improvement in the classification of these challenging classes with the addition of bio-signal inputs when a suitable DL model is used.

## Figures and Tables

**Table 1:** Random forest and CNN classifiers performance by inputs for both individual and grouped classes. Results are the mean ± standard deviation of the F1-score and area under the receiver operating characteristic (ROC) curve for 23 subjects (leave-one-subject-out mode).

Classifier	Classifier Inputs	Individual Activity Classification F1-Score	Grouped Activity Classification F1-Score	Individual Activity Classification AUC	Grouped Activity Classification AUC
RF	ACC (C0)	77.42 ± 23.01	92.52 ± 9.61	98.63 ± 2.39	99.56 ± 0.77
	ACC + BVP (C1)	76.10 ± 22.98	92.31 ± 9.56	98.57 ± 2.48	99.63 ± 0.64
	ACC + EDA (C2)	77.95 ± 22.90	93.29 ± 10.03	98.81 ± 2.53	99.82 ± 0.43
	ACC + BVP + EDA(C3)	77.21 ± 23.32	93.22 ± 10.53	98.72 ± 2.61	99.82 ± 0.47
CNN	ACC (C0)	64.22 ± 19.20	80.65 ± 11.09	94.29 ± 4.18	96.53 ± 4.08
	ACC + BVP (C1)	69.01 ± 20.27	86.34 ± 11.16	96.54 ± 3.57	98.03 ± 3.49
	ACC + EDA (C2)	67.70 ± 22.97	83.77 ± 11.84	95.93 ± 4.34	97.54 ± 2.54
	ACC + BVP + EDA(C3)	69.89 ± 18.59	87.51 ± 9.55	96.54 ± 4.21	98.48 ± 2.24

**Table 2:** Random forest classification performance for eight individual activities.

Classifier Input	ACC (C0)		ACC + BVP (C1)		ACC + EDA (C2)		ACC + BVP + EDA (C3)	
Activity	F1-Score	AUC ROC	F1-Score	AUC ROC	F1-Score	AUC ROC	F1-Score	AUC ROC
Brisk walking	83.92 ± 21.71	98.25 ± 4.43	81.34 ± 23.17	98.17 ± 4.76	82.43 ± 22.49	98.11 ± 7.16	79.46 ± 27.67	97.98 ± 6.92
Cycling	88.24 ± 19.21	99.37 ± 1.12	87.60 ± 16.42	99.57 ± 1.00	89.19 ± 19.27	99.66 ± 0.79	90.44 ± 15.85	99.70 ± 0.86
Jogging	65.60 ± 31.88	97.66 ± 3.56	63.98 ± 32.15	97.74 ± 3.38	65.15 ± 32.33	97.84 ± 3.40	65.11 ± 31.19	97.68 ± 3.54
Lying	83.22 ± 16.56	99.10 ± 1.75	80.27 ± 18.50	98.96 ± 2.06	80.71 ± 20.08	99.11 ± 1.75	79.38 ± 21.29	99.01 ± 1.97
Running	59.69 ± 35.09	98.44 ± 3.01	68.54 ± 34.27	98.41 ± 2.77	60.49 ± 33.69	98.57 ± 2.66	69.45 ± 33.91	98.54 ± 2.60
Stairs	85.97 ± 11.81	99.08 ± 1.50	85.66 ± 11.63	99.11 ± 1.40	89.65 ± 11.00	99.66 ± 0.87	89.01 ± 12.01	99.64 ± 0.92
Standing	74.00 ± 22.10	97.89 ± 2.34	72.46 ± 20.14	97.57 ± 3.09	74.32 ± 21.17	98.09 ± 2.48	74.24 ± 18.28	97.86 ± 2.91
Walking	79.36 ± 25.77	99.14 ± 1.36	78.93 ± 27.53	99.07 ± 1.42	81.69 ± 23.20	99.44 ± 1.17	80.58 ± 26.39	99.38 ± 1.17

**Table 3:** CNN classification performance for eight individual activities.

Classifier Input	ACC (C0)		ACC + BVP (C1)		ACC + EDA (C2)		ACC + BVP + EDA (C3)	
Activity	F1-Score	AUC ROC	F1-Score	AUC ROC	F1-Score	AUC ROC	F1-Score	AUC ROC
Brisk walking ^3^	65.78 ± 19.71	95.18 ± 4.23	65.06 ± 18.64	95.29 ± 3.61	64.89 ± 20.18	94.93 ± 5.70	68.72 ± 18.55	95.83 ± 3.98
Cycling ^123^	67.53 ± 16.60	95.39 ± 3.48	78.94 ± 14.67	98.11 ± 2.24	75.49 ± 16.54	97.41 ± 2.75	80.54 ± 12.96	98.32 ± 2.26
Jogging ^23^	55.53 ± 27.20	92.38 ± 4.08	56.44 ± 25.02	95.65 ± 3.57	60.72 ± 23.21	96.53 ± 2.36	58.77 ± 23.03	96.09 ± 3.55
Lying ^123^	73.75 ± 17.19	97.28 ± 3.49	81.05 ± 20.30	98.66 ± 2.13	76.99 ± 18.89	97.75 ± 3.71	80.11 ± 18.34	98.48 ± 2.43
Running	51.10 ± 21.84	95.37 ± 3.02	55.61 ± 19.28	95.77 ± 3.47	56.35 ± 18.33	95.42 ± 3.86	54.73 ± 20.11	94.76 ± 7.29
Stairs ^123^	75.19 ± 9.22	96.23 ± 4.01	80.52 ± 12.89	97.37 ± 2.98	78.81 ± 12.67	96.48 ± 5.13	81.68 ± 13.39	98.07 ± 3.12
Standing ^13^	66.94 ± 16.62	96.24 ± 2.91	74.36 ± 17.44	97.54 ± 3.36	69.63 ± 18.82	96.49 ± 3.39	73.08 ± 18.13	97.67 ± 2.73
Walking ^3^	57.95 ± 25.24	91.32 ± 8.24	60.08 ± 24.99	92.92 ± 7.21	58.72 ± 25.11	92.45 ± 7.80	61.52 ± 24.23	93.87 ± 8.35

^1^ Indicates significant improvement with added BVP. ^2^ Indicates significant improvement with added EDA. ^3^ Indicates significant improvement with added BVP and EDA.

**Table 4:** RF classification performance for grouped, stairs, and cycling activities.

Classifier Input	ACC (C0)		ACC + BVP (C1)		ACC + EDA (C2)		ACC + BVP + EDA (C3)	
Activity	F1-Score	AUC ROC	F1-Score	AUC ROC	F1-Score	AUC ROC	F1-Score	AUC ROC
Low-intensity *	96.36 ± 4.58	99.85 ± 0.27	95.46 ± 5.58	99.89 ± 0.24	96.66 ± 4.28	99.93 ± 0.14	96.67 ± 4.19	99.94 ± 0.12
Medium-intensity	92.96 ± 8.11	99.50 ± 0.80	93.05 ± 10.45	99.57 ± 0.54	92.96 ± 11.75	99.86 ± 0.22	91.86 ± 16.57	99.86 ± 0.25
High-intensity	99.33 ± 2.31	100.0 ± 0.00	99.39 ± 2.22	100.0 ± 0.00	99.30 ± 2.30	100.0 ± 2.28 × 10^−5^	99.38 ± 2.22	100.0 ± 1.14 × 10^−3^
Cycling	87.87 ± 20.91	99.41 ± 1.10	87.18 ± 17.48	99.60 ± 0.96	89.06 ± 19.86	99.64 ± 0.91	89.93 ± 17.24	99.68 ± 0.98
Stairs	86.09 ± 12.11	99.06 ± 1.67	86.46 ± 12.08	99.12 ± 1.45	88.48 ± 11.95	99.69 ± 0.86	88.24 ± 12.46	99.62 ± 1.01

* Low-intensity: lying/standing; medium-intensity: brisk walking/walking; high-intensity: jogging/running.

**Table 5:** CNN classification performance for grouped, stairs, and cycling activities.

Classifier Input	ACC (C0)		ACC + BVP (C1)		ACC + EDA (C2)		ACC + BVP + EDA (C3)	
Activity	F1-Score	AUC ROC	F1-Score	AUC ROC	F1-Score	AUC ROC	F1-Score	AUC ROC
Low-intensity *^13^	85.76 ± 8.74	96.66 ± 3.30	91.89 ± 8.06	97.55 ± 1.68	88.27 ± 7.83	96.98 ± 2.53	91.70 ± 7.95	98.15 ± 1.53
Medium-intensity ^13^	82.61 ± 9.68	92.89 ± 2.88	86.37 ± 9.35	97.53 ± 3.06	83.59 ± 12.22	96.07 ± 3.95	87.81 ± 8.36	97.60 ± 2.31
High-intensity	95.16 ± 8.79	99.66 ± 0.98	94.65 ± 11.50	99.55 ± 1.44	94.54 ± 12.12	99.53 ± 1.44	95.89 ± 7.57	99.67 ± 1.03
Cycling ^123^	65.07 ± 18.14	97.52 ± 9.11	78.76 ± 13.98	98.29 ± 2.77	74.82 ± 15.82	98.29 ± 5.29	80.63 ± 13.24	99.13 ± 3.27
Stairs ^13^	74.64 ± 10.11	95.90 ± 4.13	80.05 ± 12.93	97.21 ± 3.74	77.65 ± 11.20	96.83 ± 4.23	81.51 ± 10.62	97.87 ± 3.05

^1^ Indicates significant improvement with added BVP. ^2^ Indicates significant improvement with added EDA. ^3^ Indicates significant improvement with added BVP and EDA. * Low intensity: lying/standing; medium-intensity: brisk walking/walking; high-intensity: jogging/running.

## Data Availability

The raw data supporting the conclusions of this article will be made available by the authors upon reasonable request.

## Supplementary Materials

The following supporting information can be downloaded at https://www.mdpi.com/article/10.3390/s24103005/s1, Table S1: Classifier evaluation metrics: Matthews Correlation Coefficient, Log-loss, and Cohen’s Kappa Score. Table S2: McNemar Test results, comparing fused CNN models to accelerometer-only CNN models in classifying individual activities (p-value). Table S3: McNemar Test results, comparing fused CNN models to accelerometer-only CNN models in classifying grouped activities (p-value). Table S4: Wilcoxon Rank Sum Test results at the 5% significance level, indicating individual activity classification error improvement of fused CNN models with respect to stairs class. Only activities which are improved significantly by at least one classifier are shown. Table S5: Wilcoxon Rank Sum Test results at the 5% significance level, indicating individual activity classification error improvement of fused CNN models with respect to cycling class. Only activities which are improved significantly by at least one classifier are shown. Table S6: Wilcoxon Rank Sum Test results at the 5% significance level, indicating grouped activity classification error improvement of fused CNN models with respect to stairs class. Only activities which are improved significantly by at least one classifier are shown. Table S7: Wilcoxon Rank Sum Test results at the 5% significance level, indicating grouped activity classification error improvement of fused CNN models with respect to cycling class. Only activities which are improved significantly by at least one classifier are shown. Table S8: Random forest data set features.
