# Supplementary material for: Human Activity Recognition Algorithm with Physiological and Inertial Signals Fusion: Photoplethysmography, Electrodermal Activity, and Accelerometry
Source: Sensors (Basel). 2024 May 9;24(10):3005. doi: 10.3390/s24103005 (PMC11124986; doi:10.3390/s24103005)
Supplement: Supplementary file 1 [file sensors-24-03005-s001.zip › sensors-2935500-supplementary.pdf]

Human Activity Recognition Algorithm with Physiological and Inertial signals Fusion: Photoplethysmography, Electrodermal Activity, and Accelerometry (Supplemental Material)

Justin Gilmore , Mona Nasseri

Table S1. Classifier evaluation metrics: Matthews Correlation Coefficient, Log-loss, and Cohen's Kappa Score.

		Individual Activity Classification			Grouped Activity Classification		
**Classifier**	**Classifier Inputs**	**MCC**	**Log-loss**	**Kappa**	**MCC**	**Log-loss**	**Kappa**
RF	ACC	0.789	0.498	0.777	0.927	0.186	0.924
	ACC+BVP	0.778	0.508	0.767	0.926	0.182	0.922
	ACC+EDA	0.794	0.451	0.782	0.934	0.143	0.930
	ACC+BVP+EDA	0.792	0.471	0.779	0.933	0.151	0.929
CNN	ACC	0.619	0.963	0.609	0.787	0.467	0.783
	ACC+BVP	0.687	0.980	0.676	0.818	0.397	0.814
	ACC+EDA	0.657	0.971	0.647	0.819	0.436	0.814
	ACC+BVP+EDA	0.689	0.942	0.680	0.838	0.383	0.834

Table S2. McNemar Test results, comparing fused CNN models to accelerometer-only CNN models in classifying individual activities (*p*-value).

Classifier Inputs	Brisk-walking	Cycling	Jogging	Lying	Running	Stairs	Standing	Walking
ACC+BVP	0.045	1.21e-15	0.413	1.46e-5	0.110	5.85e-7	4.75e-6	0.119
ACC+EDA	0.843	4.88e-8	1.47e-5	0.047	0.170	2.19e-4	0.173	0.804
ACC+BVP+EDA	3.64e-5	1.07e-16	4.95e-5	1.27e-11	0.760	1.04e-12	2.68e-12	3.95e-7

Table S3. McNemar Test results, comparing fused CNN models to accelerometer-only CNN models in classifying grouped activities (*p*-value).

Classifier Inputs	Stand/Lying (low-intensity)	Brisk Walk/Walking (med-intensity)	Jog/Running (high-intensity)	Cycling	Stairs
ACC+BVP	2.43e-5	1.49e-5	1.0	8.62e-40	3.24e-8
ACC+EDA	1.0	0.622	0.004	4.13e-17	0.526
ACC+BVP+EDA	1.08e-7	1.93e-13	0.250	4.83e-44	1.24e-6

Table S4. Wilcoxon Rank Sum Test results at the 5% significance level, indicating individual activity classification error improvement of fused CNN models with respect to stairs class. Only activities which are improved significantly by at least one classifier are shown.

Classifier Inputs	Standing	Walking
ACC+BVP	0.023	0.082
ACC+EDA	0.633	0.516
ACC+BVP+EDA	0.001	0.041

Table S5. Wilcoxon Rank Sum Test results at the 5% significance level, indicating individual activity classification error improvement of fused CNN models with respect to cycling class. Only activities which are improved significantly by at least one classifier are shown.

Classifier Inputs	Lying	Standing
ACC+BVP	3.70e-4	0.044
ACC+EDA	0.252	0.005
ACC+BVP+EDA	2.30e-4	0.007

Table S6. Wilcoxon Rank Sum Test results at the 5% significance level, indicating grouped activity classification error improvement of fused CNN models with respect to stairs class. Only activities which are improved significantly by at least one classifier are shown.

Classifier Inputs	Standing/Lying (low-intensity)
ACC+BVP	0.009
ACC+EDA	0.756
ACC+BVP+EDA	0.009

Table S7. Wilcoxon Rank Sum Test results at the 5% significance level, indicating grouped activity classification error improvement of fused CNN models with respect to cycling class. Only activities which are improved significantly by at least one classifier are shown.

Classifier Inputs	Standing/Lying (low-intensity)	Brisk Walking/Walking(med-intensity)
ACC+BVP	7.10e-4	0.068
ACC+EDA	0.084	0.070
ACC+BVP+EDA	4.50e-4	0.022

Table S8. Random forest data set features.

	Tri-axial accelerometer	BVP	EDA
**Time-domain**	Shannon entropy, log energy entropy, average power, mean, minimum (min), maximum (max), standard deviation (std) , variance, IQR, skewness, kurtosis, percentiles (10, 20, 25, 50, 75, and 90), peak std, correlation (xx, xz, and yz), angle with horizon in degrees (mean, min, max, std, IQR), difference between axis amplitude (xy, xz, yz), signal mean amplitude	Mean, min, max, std, variance, IQR, skewness, kurtosis, Shannon entropy, log energy entropy, average power, percentiles (10, 20, 25, 50, 75, and 90), peak std	Mean, min, max, std, variance, skewness, kurtosis, Shannon entropy, log energy entropy
**Frequency-domain**	Highest frequency, dominant frequency, normalized power (0.5, 1, 2, 3, 4, and 5)Hz	Dominant frequency, power in band (0.4-0.57, 0.9-1.07, 1.9-2.07, and 2.9-3.07)Hz	Power in band (0.031-0.22, 0.15-0.33, 0.18-0.438, 0.25-0.5)Hz
